# Underutilization of the Emergency Department During the COVID-19 Pandemic

**DOI:** 10.5811/westjem.2020.8.48632

**Published:** 2020-09-24

**Authors:** Anthony D. Lucero, Andre Lee, Jenny Hyun, Carol Lee, Chadi Kahwaji, Gregg Miller, Michael Neeki, Joshua Tamayo-Sarver, Luhong Pan

**Affiliations:** * Kaweah Delta Medical Center, Department of Emergency Medicine, Visalia, California; † Vituity, Department of Enterprise Data Analytics, Emeryville, California; ‡ Arrowhead Regional Medical Center, Department of Emergency Medicine, Colton, California; § Swedish Edmonds Campus, Department of Emergency Medicine, Edmonds, Washington; ¶ Good Samaritan Hospital, Department of Emergency Medicine, San Jose, California

## Abstract

**Introduction:**

The novel coronavirus 2019 (COVID-19) pandemic in the United States (US) prompted widespread containment measures such as shelter-in-place (SIP) orders. The goal of our study was to determine whether there was a significant change in overall volume and proportion of emergency department (ED) encounters since SIP measures began.

**Methods:**

This was a retrospective, observational, cross-sectional study using billing data from January 1, 2017–April 20, 2020. We received data from 141 EDs across 16 states, encompassing a convenience sample of 26,223,438 ED encounters. We used a generalized least squares regression approach to ascertain changes for overall ED encounters, hospital admissions, and New York University ED visit algorithm categories.

**Results:**

ED encounters decreased significantly in the post-SIP period. Overall, there was a 39.6% decrease in ED encounters compared to expected volume in the pre-SIP period. Emergent encounters decreased by 35.8%, while non-emergent encounters decreased by 52.1%. Psychiatric encounters decreased by 30.2%. Encounters related to drugs and alcohol decreased the least, by 9.3% and 27.5%, respectively.

**Conclusion:**

There was a significant overall reduction in ED utilization in the post-SIP period. There was a greater reduction in lower acuity encounters than higher acuity encounters. Of all subtypes of ED encounters, substance abuse- and alcohol-related encounters reduced the least, and injury-related encounters reduced the most.

## INTRODUCTION

The coronavirus disease 2019 (COVID-19) is an ongoing global crisis with far-reaching social consequences. First reported in Wuhan, China, in December 2019, COVID-19 quickly spread across that country, despite a government-mandated lockdown of Wuhan on January 23, 2020. 1–4 By the time the World Health Organization (WHO) officially recognized the pandemic status of COVID-19 on March 11, 2020, there were over 118,000 confirmed cases globally and over 4,200 deaths.^5^ As of July 27, 2020, there were more than 4.2 million cases in the United States (US), with 146,546 related deaths.^6^

The large-scale social impact of COVID-19 has not been seen since the influenza pandemic of 1918 when non-pharmaceutical interventions – banning large public gatherings, school closures, and voluntary quarantine of diseased households – were most notably implemented on a large scale to decrease disease transmission.^7^–^8^ The disproportionally high mortality rate due to COVID-19 in Spain and Italy is partly attributed to those countries’ healthcare systems becoming quickly overwhelmed by the volume of critical patients. Specifically, these countries experienced severe shortages of intensive care unit beds and ventilators.^9^–^13^ The impact of the virus was projected to also overwhelm the US healthcare system, which resulted in widespread implementation of shelter-in-place (SIP) restrictions.^14^ As early as March 19, 2020, state governments within the US began issuing SIP directives with the goal to “flatten the curve,” a term used by the Centers for Disease Control and Prevention (CDC) referring to strategies to slow the rate of disease progression to avoid overwhelming the healthcare system.^15^–^16^

Since the implementation of SIP directives, there have been reports of a significant drop in emergency department (ED) volumes by 40–50%.^17^ News media have reported alarming reductions in ED visits related to acute coronary syndrome and cerebral vascular accidents.^17^–^20^ Recent studies have corroborated these reports from the media regarding reductions in non-COVID-19 related ED visits.^21^–^25^ Similar findings in Europe and China have also been reported, with the hypothesis that fear of coming to the hospital may be preventing patients from seeking care, especially those experiencing less severe symptoms.^26^–^29^ A recent poll from the American College of Emergency Physicians (ACEP) aligns with these suspicions, reporting that nearly a third of American adults have deferred medical care to avoid contracting COVID-19.^30^ A high proportion of those polled (73%) were concerned about burdening the healthcare system or not receiving adequate care during pandemic conditions.^31^ This may be contributing to “excess deaths without COVID-19,” which the CDC defines as the rise in non-COVID-19 related deaths beyond what would be expected.^32^ In fact, a recent, single-center US study showed that 0% of stroke patients who arrived to the ED following SIP orders were within the window for tissue plasminogen activator, which is much lower than the national average of 3.71%.^33^,^34^ Consequently, ACEP is urging providers to reach out to the public to avoid further delays in care.^35^

To date, there is limited literature assessing the impact of the current COVID-19 pandemic on ED volumes across various encounter types in the US. An accurate assessment of the collateral effects beyond COVID-19 infection is crucial to guiding current and future public health management. We sought to determine whether there was a significant change in overall volume and proportion of various types of encounters in the ED since COVID-19 containment measures began. This study was an epidemiological analysis using retrospective billing data across 141 EDs comparing numbers before and after the first SIP orders in the US on March 16, 2020.^36^ We subdivided ED encounters into four categories (non-emergent; emergent-primary care treatable; emergent-preventable; and emergent). Our analysis also included a separate categorization of mental health, alcohol, substance abuse, and acute injury-related encounters, in hopes of shedding light on possible behavior-driven emergencies during pandemic circumstances.

**Population Health Research Capsule**

What do we already know about this issue?

*The coronavirus disease 2019 (COVID-19) pandemic resulted in widespread social distancing measures, leading to concern for decreased emergency department (ED) visits*.

What was the research question?

Was there a change in overall volume and proportion of various types of ED visits following shelter-in-place (SIP) orders?

What was the major finding of the study?

*Total ED volumes decreased, with the greatest reduction in low acuity visits and the least in drug- and alcohol-related visits*.

How does this improve population health?

*This study shows the link between SIP orders and ED use during the initial weeks of the COVID-19 pandemic*.

## METHODS

### Study Design and Data Source

This study was approved by the Arrowhead Regional Medical Center Institutional Review Board. Using a retrospective, observational, cross-sectional design, we analyzed ED log and billing data associated with a physician services billing company. Select demographic information provided by hospital medical record data was used to supplement the ED log data, in addition to coded billing data on primary diagnoses and procedures. Each patient billing record could hold up to four diagnosis codes and four procedure codes. Charges encompassed the physician services billing portion of the patient ED encounter, not the hospital billing charges. Dates where SIP orders were instituted make up the pre- and post-SIP periods (see Appendix A).^15^ For the purposes of this study, pre- and post-SIP periods were determined by state-specific dates in the state in which the hospital was located.

The study data set consisted of billing data from January 1, 2017–April 20, 2020, which encompassed 26,223,438 encounters across 141 EDs in 16 states within the US. Hospitals represented seven of the 10 Centers for Medicare and Medicaid Services (CMS) regions. Because the study data set is at the encounter level, patients could be represented multiple times within the data set if they returned to the ED for care. Patient characteristics, such as gender, age, hospital disposition, type of provider seen during encounter (physician or advanced practice provider), and Emergency Severity Index (ESI) level for the encounter are presented in Table 1. The ESI is a five-level ED triage algorithm that provides clinical stratification on the basis of acuity and resource needs, with level one being the most urgent and level five the least urgent.

Table 2 shows hospital characteristics of the 141 EDs included in the analysis. Hospital characteristics, including state, ownership, urban/rural, and teaching status, were taken from the 2018 American Hospital Association Annual Survey. Hospital characteristics were null if survey data was not submitted. Hospital ownership typology was standardized from 14 to nine categories for ease of computations (see Appendix B). Hospitals were allowed to self-select the subcategory type of organization (eg, non-federal government; non-government, not-for-profit; investor-owned, for-profit; federal government) that best described their hospital’s policies and operations.

Categorization of emergent and non-emergent ED encounters was done using the New York University (NYU) ED visit algorithm (EDA).^37^–^39^ Per the NYU EDA methodology, we used the diagnosis weights to calculate the number of emergent, emergent-preventable, emergent-primary care treatable, and non-emergent encounters per day per site, in addition to the “alcohol,” “drug,” “injury,” “psychiatric,” and “unclassified” diagnostic categories.

The NYU EDA sets specific criteria for each category of ED encounter regarding how emergent the encounter is. **Emergent** care represents care for an acute condition where ED care was required. **Emergent-preventable** care represents care where ED care was required for an acute exacerbation but could have been treated or prevented with ready access to primary care. **Emergent-primary care treatable** is care that should be administered within 12 hours of presentation, but care could have been safely and effectively delivered within a primary care setting. **Non-emergent** care represents an encounter where care was not needed for at least 12 hours. For the NYU EDA diagnostic categories, **Alcohol** represents care for alcohol intoxication-related care. **Substance Abuse** represents care for non-alcohol substance use (eg, opioid, cannabis, sedatives) intoxication or complications. **Injury** represents care for trauma, such as accidents and lacerations. **Mental Health** represents care for various psychiatric disorders (eg, schizophrenia, bipolar, major depressive, and intentional self-harm). **Unclassified** represents care for diagnoses that could not otherwise be categorized per above.

We used hospital discharge dispositions from billing data to ascertain admission status. ED encounters with admit or transfer discharge disposition were counted as a hospital admission. Hospital admission was limited to patients who presented through the ED and did not include directly admitted patients.

### Data Analysis

Descriptive statistics of patient and hospital characteristics are presented in Table 1 and Table 2, respectively. Percentages represent the proportion of ED encounters that fell within each respective pre-SIP or post-SIP category. Using a random effects generalized least squares (GLS) modeling approach, we ran regression analyses using Stata, version 16.1 (StataCorp, College Station, TX). A GLS approach was used to control for correlations in utilization patterns within hospitals and across time, ie, seasonality. In addition, to correct for known utilization patterns in ED encounters, we averaged encounters by site per month and per day of week to create an “expected” number of encounters. The dependent variable was then calculated as percent variance from the expected encounter volume per site, calculated as [(Observed – Expected) / Expected]. The GLS regression included the intercept and coefficient for SIP. In the GLS results, we interpreted positive coefficients as the percent increase compared to pre-SIP expected levels, whereas we interpreted negative coefficients as the percent decrease compared to pre-SIP expected levels (Table 3).

## RESULTS

### Characteristics of Study Subjects

The data shows that there was a shift in the types of patients who used the ED in the pre- and post-SIP periods. Women and patients in the 35–64 and 65+ age groups made up the majority of patient encounters overall. The percentage of pediatric encounters (birth–18 years old) decreased from 16.2% to 8.4% in the post-SIP period. The distribution of patients across ESI levels demonstrated a bell-shaped distribution both pre- and post-SIP periods, where the majority of cases had ESI levels between 2–4. However, ED encounters with ESI levels 1–3 were proportionally higher in the post-SIP period. There was an increase in the proportion of patients who had an admit or transfer disposition following an initial ED encounter in the post-SIP period, 23.6%, vs 20.3% in the pre-SIP period.

Of the seven CMS regions represented in our study data, the largest proportion of ED encounters came from Region 9 (San Francisco) with 76.8% of total patient encounters for the study period. The majority of patient encounters occurred in hospitals that were minor teaching (49.1%) or non-teaching (19.0%) hospitals in urban locations. Hospitals that were non-profit, either religious-affiliated (15.6%) or other non-profit (33.9%), represented the plurality of patient encounters with the remaining encounters spread relatively evenly across county (7.1%), for-profit (8.7%), and hospital district (4.9%) hospitals. The remaining 29.8% of patient encounters occurred in hospitals that did not report hospital organization type.

### ED Encounters and Shelter-in-Place

**Figure 1:**
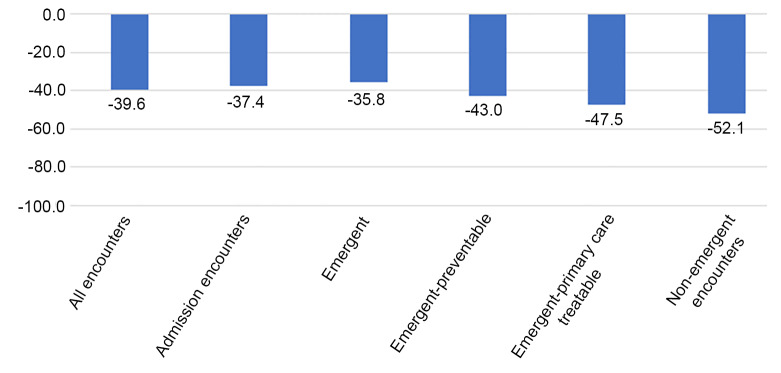
Percent change from pre-shelter in place.

**Figure 2:**
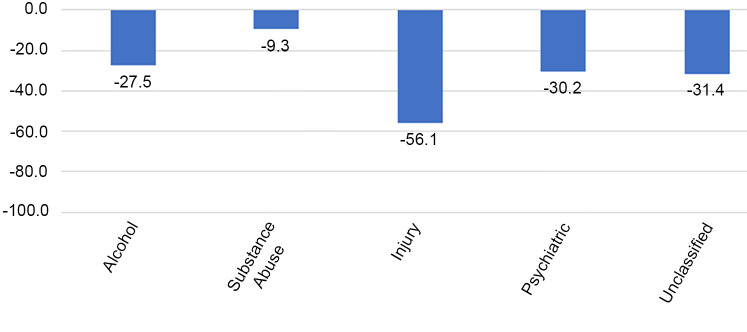
Percent change from pre-shelter in place.

There was a significant reduction in the number of ED encounters in the post-SIP period. Overall, there was a 39.6% decrease (95% confidence interval (CI). −40.8%, −38.5%) in all ED encounters compared to what would have been expected in the study period. The greatest decrease was seen in the non-emergent encounters (−52.1%), followed by emergent-primary care treatable encounters (−47.5%), emergent-preventable encounters (−43.0%), and then emergent encounters (−35.8%) (Table 3, Figure 1). Hospital admissions saw an overall decrease of 37.4% (95% CI, −38.4%, −36.5%) compared to pre-SIP period. The group of diagnoses that saw the biggest decrease in the post-SIP period was injury with a 56.1% decrease compared to the pre-SIP period (Figure 2). Encounters for substance abuse and alcohol-related treatment saw the smallest reduction, at 9.3% and 27.5%, respectively (Figure 2).

## DISCUSSION

Our analysis demonstrates that, after SIP orders were implemented, there was a 39.6% reduction in overall ED utilization. There are several, well-publicized theories as to why such a pronounced drop in volume occurred. One reason might be a true reduction in disease burden, especially a decline in traumatic injuries, due to the SIP order. However, other factors certainly contributed. An April 2020 ACEP poll suggested that public fear of potentially contracting COVID-19 from a hospital visit deterred patients from visiting EDs for conditions that they would have sought ED treatment under non-pandemic circumstances.^30^ Additionally, the public health campaign to discourage “over-burdening the healthcare system” may have also contributed to the overall decrease in the frequency of ED visits.^31^

The proportion of patients admitted or transferred from the ED was higher post-SIP (23.6%) compared to pre-SIP (20.3%). Additionally, there was an increase in the proportion of patients with higher acuity ESI levels presenting to the ED post-SIP. The proportion of ESI levels 1, 2, and 3 increased with respect to ESI levels 4 and 5 post-SIP. This would suggest that the patients presenting to the ED post-SIP generally had self-selected for more serious conditions as compared to pre-SIP, and more of the “missing” visits were associated with lower acuity complaints.

There were also differences in regard to the age of patients presenting to the ED before and after the SIP. The proportion of pediatric patients (birth–18 years old) presenting to the ED declined from 16.2% pre-SIP to 8.4% post-SIP. Conversely, the proportion of older patients (>35 years old) presenting to the ED increased from 61.5 % pre-SIP to 68.8% post-SIP. It would be difficult to determine exactly why such trends were noted. One possibility is that a parent’s weighing of the risk exposure to COVID-19 in the ED vs the benefit of being evaluated, as it relates to the decision to bring their child to the ED, is different than that of an independent adult deciding on their own care. Also, despite recent literature suggesting a potential rise in non-accidental trauma due to increased stressors at home during the pandemic, non-accidental trauma remains difficult to identify and often is under-reported.^40^ Another possibility is that older patients tend to present more often with higher acuity medical conditions, who may be less likely to forego ED visits.^41^–^42^

Our study found that all categories of ED encounters set forth by the NYU EDA experienced a significant reduction post-SIP compared to pre-SIP. The reduction seen in the most emergent group (emergent-ED care needed-not preventable) was smaller when compared to all other categories. Furthermore, we found that as the acuity levels increased, there was less of a reduction of ED utilization in the post-SIP period. Despite this, the observation of a 35.8% drop in emergent encounters is a concerning finding. The long-term consequences of this large drop in emergent ED encounters is difficult to quantify, but clearly could have the potential to be far-reaching. This significant reduction in volume indicates that the most emergent patients are foregoing necessary treatments, raising concerns for an increase in overall morbidity and mortality.^32^–^34^

Interestingly, ED encounters related to substance and alcohol abuse experienced the lowest reduction in the post-SIP period. For example, substance abuse-related ED encounters dropped by only 9.3% in the post-SIP period, while alcohol-related encounters dropped by 27.5%. This effect may be explained by the previously well-documented relationship between large-scale disasters and increased drug and alcohol abuse. Studies that looked at previous large-scale disasters such as Hurricane Katrina, the 2004 Southeast Asia tsunami, and the 2001 September 11 attacks, all reported an increase in either drug or alcohol abuse.^43^–^45^ This raises the question as to whether we will see an increase in ED encounters related to drug and alcohol abuse as the COVID-19 pandemic continues to unfold.

Similarly, the 30.2% decline in visits with psychiatric diagnoses was smaller than the decline in emergent (−35.8%) and non-emergent (−52.1%) visits. Several studies suggest that depressive disorders and post-traumatic stress disorder have increased as a result of COVID-19.^46^–^47^ Perhaps any decline in baseline psychiatric visits was mitigated by an upward trend in mental health issues provoked by pandemic.

On the contrary, injury-related ED encounters experienced the greatest reduction (−56.1%) between pre- and post-SIP. We suspect this may in part be explained by the fact that injury is heavily dependent on individual behavior, and that behaviors promoted by pandemic measures have made people more cautious and less prone to experiencing injury. There may have been fewer motor vehicle accidents because people generally drove less due to SIP measures. Similarly, there may have been fewer work-related injuries due to more people working from home.^48^ Traffic and community activity reports in the US show a correlation with a drop of 48% in personal traffic and transit stations compared to baseline.^49^ A recent study in New Hampshire supports these findings, reporting a 57% decrease in trauma admissions and 80% decrease in motor vehicle accidents.^50^ Another possible explanation is that cancellations of high-risk sports may have contributed to a reduction in blunt trauma.^51^ Other studies postulated that reductions in orthopedic trauma may also be partly due to social distancing measures limiting social interactions.^52^–^53^ We suspect that reductions in injury-related ED encounters is likely a multifactorial phenomenon.

While the focus of this and several other recent studies has been on the alarming reduction of emergent cases presenting at hospitals during the post-SIP period, the other side of the coin is a reduction in non-emergent and emergent-primary care treatable encounters that are best treated outside of high-cost hospital EDs. It is likely that a large proportion of patients who would have presented to the ED as non-emergent and emergent-primary care treatable encounters chose to forego care entirely. Another research question is to what extent did those patients choose to receive care in non-acute settings, such as urgent care or primary care clinics.

While the study results have high external validity given the breadth of patient encounter data from 16 different states in the US, wider generalizability to international health systems may be limited by the particular insurance-based/fee-for-service payment system that is characteristic of the US healthcare system. Furthermore, the study data had a large proportion of encounters from the CMS Region 9, which may impact generalizability to other regions of the US.

There are several follow-up research questions that could be asked from these findings. Future studies could investigate whether inadequate access to primary care offices due to SIP-related closures affected ED utilization. Findings would have far-reaching implications on primary care preparations in anticipation of a possible “second wave” of SIP closures or future pandemic planning. Another interesting topic to explore is whether rates of substance and alcohol abuse, and any complications thereof, will increase as the COVID-19 pandemic unfolds. A future study might explore whether ED utilization was absorbed by telehealth encounters, and to what extent. Future survey studies could explore perceptions of ED care during the post-SIP period and whether there were substantial changes in behaviors, such as engagement in hazardous activities, to reduce exposure to injury and hospitalization. Additionally, the long-term impact of the pandemic on the public’s utilization of the ED for low-acuity visits should be assessed. Lastly, another important topic to explore is whether the delays in care due to not presenting to the ED correlated with an increase in morbidity and/or mortality, not directly related to COVID-19.

## CONCLUSION

There was a 39.6% reduction in all ED encounters in the post-SIP period across all ED sites. The largest proportional reduction in ED encounters came from preventable and non-emergent ED encounters that could most likely have been treated at primary care offices. However, the large reduction in emergent ED encounters may potentially have delayed treatment and increased mortality seen outside of the ED. Of the five diagnostic categories in the NYU ED algorithm, injury-related ED encounters had the greatest reduction (−56.1%). This is may be a result of less motor vehicle travel and fewer hazardous work activities that contributed to the prevention of injuries. Substance and alcohol abuse-related encounters had the lowest reduction in the post-SIP period (−9.3% and −27.5%, respectively), describing the relatively unchanging nature of these disorders in needing emergent interventions, or possibly related to increased substance use associated with the pandemic.

## Supplementary Information





## Figures and Tables

**Table 1:** Emergency department encounter distribution before and after shelter-in-place orders by patient characteristics.

	Pre-SIP encounters (n)	Pre-SIP encounters (%)	Post-SIP encounters (n)	Post-SIP encounters (%)
Gender				
Female	14,091,085	54.4	172,307	50.8
Male	11,793,299	45.6	166,747	49.9
Disposition				
Admit	4,455,299	17.2	68,775	20.3
Discharge	20,629,288	79.7	259,090	76.4
Transfer	799,797	3.1	11,189	3.3
ESI Level*				
1	159,801	0.8	2,822	1.2
2	2,697,452	14.0	38,238	16.0
3	10,164,404	52.7	129,558	54.2
4	5,614,369	29.1	60,251	25.2
5	658,951	3.4	8,131	3.4
Provider type				
Physician	18,639,401	72.0	250,972	74.0
Advanced practice provider	7,227,121	27.9	87,865	25.9
Age Group				
Age < 1	485,097	1.9	3,291	1.0
1 ≤ Age < 18	3,697,234	14.3	25,103	7.4
18 ≤ Age < 35	5,793,875	22.4	77,276	22.8
35 ≤ Age <65	6,357,256	24.5	89,196	26.3
Age > 65	9,548,938	36.9	144,113	42.5
Total	25,884,384	98.7	339,054	1.3

* ESI level is coded from 1 to 5, where 1 represents most urgent and 5 represents least urgent.

Note: Within each characteristic, total percentages may not sum up to 100 due to null values. All differences in pre- and post-SIP categories significant at p<.001 due to high sample size.

*SIP*, shelter in place; *ESI*, Emergency Severity Index.

**Table 2:** Encounter distribution by hospital characteristics.

	Pre-SIP encounters (n)	Pre-SIP encounters (%)	Post-SIP encounters (n)	Post-SIP encounters (%)
CMS region - regional office				
Region 3 - Philadelphia	709,649	2.7	5,866	1.7
Region 4 - Atlanta	425,961	1.7	2,772	0.8
Region 5 - Chicago	2,590,841	10.0	41,731	12.3
Region 6 - Dallas	1,577	0.0	396	0.1
Region 7 - Kansas City	705,385	2.7	4,459	1.3
Region 9 - San Francisco	19,874,290	76.8	263,555	77.7
Region 10- Seattle	1,576,681	6.1	20,275	6.0
AHA teaching status				
Major (2)	556,472	2.2	6,078	1.9
Minor (31)	12,714,363	49.1	170,158	50.2
Non-teaching (51)	4,900,455	18.9	68,991	20.4
AHA location				
Rural (4)	281,445	1.1	4,452	1.3
Urban (88)	17,889,845	69.1	240,775	71.0
Ownership				
Non-profit (42)	8,777,429	33.9	118,962	35.1
For-profit (12)	2,247,155	8.7	30,213	8.9
Religious (26)	4,044,370	15.6	53,300	15.7
Hospital district (6)	1,277,315	4.9	18,655	5.5
County (6)	1,825,021	7.1	24,097	7.1
Total (141)	25,884,384	98.7	339,054	1.3

* Within each characteristic, total percentages may not sum up to 100 due to null values. All differences in pre- and post-SIP categories significant at p<.001 due to high sample size.

*SIP*, shelter in place; *AHA*, American Hospital Association.

**Table 3:** Regression results.

Dependent variable	% Change compared to pre-SIP	Standard error (SE)	95% confidence interval (CI)
All encounters	−39.6	0.006	−40.8, −38.5
Admission encounters	−37.4	0.005	−38.4, −36.5
Emergent	−35.8	0.005	−36.9, −34.6
Emergent-preventable	−43.0	0.005	−43.9, −42.0
Emergent-primary care treatable	−47.5	0.003	−48.1, −46.9
Non-emergent encounters	−52.1	0.004	−52.8, −51.4
Alcohol	−27.5	0.017	−30.4, −24.6
Substance abuse	−9.3	0.020	−13.2, −5.4
Injury	−56.1	0.004	−56.9, −55.2
Psychiatric	−30.2	0.011	−32.3, −28.1
Unclassified	−31.4	0.005	−32.4, −30.5
