# Supplementary material for: Underutilization of the Emergency Department During the COVID-19 Pandemic
Source: West J Emerg Med. 2020 Sep 24;21(6):15–23. doi: 10.5811/westjem.2020.8.48632 (PMC7673895; doi:10.5811/westjem.2020.8.48632)
Supplement: Supplementary file 1 [file wjem-21-15-s001.docx]

| **Appendix A: Shelter-in-Place Order Dates by State** | |
| --- | --- |
| **State** | **Shelter-in-Place Date** |
| Arizona | 4/4/2020 |
| California | 3/19/2020 |
| Washington, DC | 4/1/2020 |
| Georgia | 4/3/2020 |
| Idaho | 3/25/202 |
| Illinois | 3/21/2020 |
| Indiana | 3/24/2020 |
| Kansas | 3/30/2020 |
| Maryland | 3/30/2020 |
| Missouri | 4/6/2020 |
| Nevada | 4/1/2020 |
| Ohio | 3/23/2020 |
| Oregon | 3/23/2020 |
| Pennsylvania | 4/1/2020 |
| Texas | 4/2/2020 |
| Washington | 3/23/2020 |
