# Supplementary material for: Underutilization of the Emergency Department During the COVID-19 Pandemic
Source: West J Emerg Med. 2020 Sep 24;21(6):15–23. doi: 10.5811/westjem.2020.8.48632 (PMC7673895; doi:10.5811/westjem.2020.8.48632)
Supplement: Supplementary file 2 [file wjem-21-15-s002.docx]

| **Appendix B: Hospital Ownership** | |
| --- | --- |
| **Control / Ownership Type** | **Standardized Ownership** |
| Nongovt. (not-for-profit) - Other | Non-profit |
| Investor-owned; for-profit - Corporation | For-profit |
| Nongovt. (not-for-profit) - Church Operated | Church |
| Govt. (federal) - Veterans' Affairs | Military/Federal |
| Govt. (federal) - Public Service Other | Military/Federal |
| Department of Defense (federal) | Military/Federal |
| Govt. (non federal) - State | State |
| Investor-owned; for-profit - Partnership | For-profit |
| Govt. (non federal) - Hospital district | Hospital District |
| Investor-owned; for-profit - Individual | For-profit |
| Govt. (non federal) - County | County |
| Govt. (non federal) - City-county | City-County |
| Govt. (non federal) - City | City |
| Govt. (federal) - Public Health Indian Service | Military/Federal |
